# Label-Free and Sensitive Fluorescent Detection of Sequence-Specific Single-Strand DNA Based on S1 Nuclease Cleavage Effects

**DOI:** 10.1371/journal.pone.0108401

**Published:** 2014-10-06

**Authors:** Zheng Guan, Jinchuan Liu, Wenhui Bai, Zhenzhen Lv, Xiaoling Jiang, Shuming Yang, Ailiang Chen, Guiyuan Lv

**Affiliations:** 1 Institute of Quality Standards and Testing Technology for Agro-products, Key Laboratory of Agro-product Quality and Safety, Chinese Academy of Agricultural Sciences; Key Laboratory of Agro-food Quality and Safety, Ministry of Agriculture, Beijing, China; 2 Institute of Materia Medica, Zhejiang Chinese Medical University, Hangzhou, China; Universität Stuttgart, Germany

## Abstract

The ability to detect sequence-specific single-strand DNA (ssDNA) in complex, contaminant-ridden samples, using a fluorescent method directly without a DNA extraction and PCR step could simplify the detection of pathogens in the field and in the clinic. Here, we have demonstrated a simple label-free sensing strategy to detect ssDNA by employing its complementary ssDNA, S1 nuclease and nucleic acid fluorescent dyes. Upon clearing away redundant complementary ssDNA and possibly mismatched double strand DNA by using S1 nuclease, the fluorescent signal-to-noise ratio could be increased dramatically. It enabled the method to be adaptable to three different types of DNA fluorescent dyes and the ability to detect target ssDNA in complex, multicomponent samples, like tissue homogenate. The method can distinguish a two-base mismatch from avian influenza A (H1N1) virus. Also, it can detect the appearance of 50 pM target ssDNA in 0.5 µg·mL^−1^ Lambda DNA, and 50 nM target ssDNA in 5 µg·mL^−1^ Lambda DNA or in tissue homogenate. It is facile and cost-effective, and could be easily extended to detect other ssDNA with many common nucleic acid fluorescent dyes.

## Introduction

Single strand DNA or RNA analysis plays an important role in the molecular biology, medicine and diagnosis. This has driven the development of various methods to detect sequence-specific DNA or RNA with high sensitivity and specificity. Most traditional methods for ssDNA and RNA detection employed PCR and agarose electrophoresis, or probe hybridization and fluorescent detection. Although these methods have been used successfully to detect ssDNA sensitively, many of them are often subjected to non-specific amplification or hybridization as well as complicated and tedious work. Recently, some nanomaterials including gold nanoparticles, quantum dots, carbon nanotubes and graphene were used for fluorescence assays of ssDNA and RNA [1]–[8]. However, these methods have also some limitations such as base selectivity, insufficient sensitivity and high-cost, especially for sequence-specific ssDNA and RNA.

To address these limitations, in this study, a highly sensitive and cost-effective approach for sequence-specific ssDNA detection was developed by using complementary single-strand DNA, S1 nuclease and nucleic acid fluorescent dyes. In the proposed method, an S1 nuclease-mediated ssDNA cleavage mechanism [9]–[11] was introduced to increase the assay sensitivity by eliminating nonspecific fluorescence effect of redundant complementary ssDNA sequences and mismatches. Furthermore, the fluorescence intensity with different sequence, different lengths of oligonucleotides, and their hybrid products after S1 nuclease-mediated cleavage were also studied. To test the universality and sensitivity of the method, three different types of DNA fluorescent dyes and three specific virus ssDNA sequences were investigated and the linear ranges were compared. Moreover, in order to illustrate its practical use, 4% pork muscle tissue homogenate was used to simulate the reality, and the targets in the tissue homogenate have been directly detected without a DNA extraction and PCR step. Given its universality, easy operation, cost-effectivity, high sensitivity and specificity, this strategy provides a new alternative for the detection of ssDNA in the field and in the clinic.

## Materials and Methods

### Ethics Statement

The tissue homogenate is made from pork, which was purchased from Beijing Chaoshifa Chain Store Co., Ltd. No live animal was used in this study.

### Materials and reagents

Oligonucleotides was synthesized and purified through HPLC by Sangon Biotechnology Co., Ltd. (Shanghai, China). The sequences are listed in Table 1. The fluorescent dyes SYBR Green II (10,000× concentrated), SYBR Gold (10,000× concentrated), and PicoGreen dsDNA Reagent and Kits (200× concentrated, including 100 µg·mL^−1^ Lambda DNA standard) were purchased from Invitrogen (CA, USA). S1 nuclease was obtained from Fermentas (Beijing, China) and Takara Biotechnology Co., Ltd (Dalian, China). 96 well black polystyrene microplate (12 strips of 8 wells), MaxiSorp, were purchased from NUNC (Roskilde, Denmark). DNA hybridization buffer (HB) was prepared using Milli-Q water: 50 mM Tris-HCl buffer (pH 8.0), 0.9% (W/V) NaCl. Synthesized ssDNA dry powder samples were dissolved in Milli-Q water to obtain 10 µM stock solutions. Then it was diluted gradually from 64 nM to 50 pM ssDNA standard solutions. All other reagents were of analytical grade.

**Table 1:** Synthesized oligonucleotide sequences used in the experiments.

DNA	DNA base sequence	Total bases	A/T number	C/G number
H1N1-ssDNA	5′-CTACCATGCGAACAATTCAACCGACACTGTT-3′	31	17	14
C-H1N1-ssDNA	5′-AACAGTGTCGGTTGAATTGTTCGCATGGTAG-3′	31	17	14
CaM-ssDNA	5′-CATCGTTGAAGATGCCTCTGCCG-3′	23	10	13
C-CaM-ssDNA	5′-CGGCAGAGGCATCTTCAACGATG-3′	23	10	13
HCV-ssDNA	5′-TAATGAGGGCTGCGGGTGGG-3′	20	7	13
C-HCV-ssDNA	5′-CCCACCCGCAGCCCTCATTA-3′	20	7	13
H1N1-1-G	5′-AACAGTGTCGGTTGAATTGTTCG**G**ATGGTAG-3′	31	17	14
H1N1-1-A	5′-AACAGTG**A**CGGTTGAATTGTTCGCATGGTAG-3′	31	17	14
H1N1-2-CG	5′-AACAGT**C**TCGGTTGAATTGTTCG**G**ATGGTAG-3′	31	17	14
H1N1-2-AT	5′-AACAGTG**A**CGGTTGA**T**TTGTTCGCATGGTAG-3′	31	17	14
H1N1-3-CCG	5′-AACAGT**C**TCGGTT**C**AATTGTTCG**G**ATGGTAG-3′	31	17	14
H1N1-3-ATT	5′-AACAGTG**A**CGGTTGA**T**TTGTTCGC**T**TGGTAG-3′	31	17	14
A5, A10, A15, A20, A25, A30	All of the bases are adenine and the number is adenine number.			
T5, T10, T15, T20, T25, T30	All of the bases are thymine and the number is thymine number.			
G10	All of the bases are guanosine and the number is guanosine number.			
C10	All of the bases are cytosine and the number is cytosine number.			

C-H1N1-ssDNA, C-CaM-ssDNA, C-HCV-ssDNA: complementary DNA of H1N1, CaM, HCV, respectively. H1N1-1-G, H1N1-1-A, H1N1-2-CG, H1N1-2-AT, H1N1-3-CCG, H1N1-3-ATT; complementary DNA of H1N1 with one to three base mismatches, “-G, -A, -CG, -AT, -CCG, -ATT” are the variations.

### Parallel analysis of different oligonucleotides lengths and sequences

Oligonucleotides (T5, T10, T15, T20, T25, T30, A5, A10, A15, A20, A25, A30, C10, G10) were used for this analysis. Samples were hybridized in 100 µL HB (containing 20 µL 0.5 µM target ssDNA HB solution and its complementary ssDNA HB solution twice as much) by incubating at room temperature for 5 min [12]. Then 90 µL S1 nuclease solution (30 U, prepared according to the instruction: 0.1 µL S1 nuclease per 30 µL 1× reaction buffer, purchased from Fermentas, Beijing, China) was added and incubated at room temperature for 30 min. After that, 10 µL 20× PicoGreen was added and incubated for another 5 min. Then the fluorescent intensities were recorded using an Infinite F200 multifunction microplate reader (TECAN Austria GmbH, Grödig, Austria). The excitation (Ex) and emission (Em) wavelengths were 480 nm and 520 nm, respectively, and the slit width of filters both were 10 nm. All experiments were repeated three times.

### Adaptability study by using three different types of DNA fluorescent dyes

To observe the universality and sensitivity of our approach, we used three different types of fluorescent DNA-dyes to react with three different virus ssDNA: avian influenza H1N1 virus (H1N1-ssDNA), cauliflower mosaic virus (CaM-ssDNA) and hepatitis C virus (HCV-ssDNA) (Table 1). Besides that, the fluorescence intensity with different lengths, different A/T number in oligonucleotides (hepatitis C virus, cauliflower mosaic virus, and H1N1 virus), and their hybrid products were also studied. The reaction system is the same as described above. One group was hybridized with complementary ssDNA solution, and the other group was hybridized without complementary ssDNA, the same volume HB was used to replace the complementary ssDNA solution. Moreover, a system blank (without DNA) has also been detected as a background fluorescent intensity control. After staining with the DNA-dye, the fluorescent spectra and intensities were obtained by an LS-55 Fluorescence Spectrometer (Perkin-Elmer, Connecticut, USA). The slit width of filters both were 10 nm. The Ex and Em wavelengths were as follows:

SYBR Green II, Ex 497 nm, Em 520 nm;
SYBR Gold, Ex 495 nm, Em 537 nm;
PicoGreen, Ex 480 nm, Em 520 nm.

### Target sequence-specificity measurements

All assay conditions were the same as described in “parallel analysis” section, except the concentrations of targets: H1N1, CaM, HCV virus ssDNA (Table 1) standard solutions (0, 0.05, 0.5, 1, 2, 4, 8, 16, 32, 64 nM) and additional 5 ng·mL^−1^ Lambda DNA standard in every well as external dsDNA contamination, for their linear ranges in potential interference surroundings. The fluorescent intensities were recorded using an Infinite F200 multifunction microplate reader. Their linear ranges were acquired by deducting Lambda DNA standard background fluorescence first.

In addition, HCV (50 pM and 50 nM) was selected as an example to record the detection limit of this method, while a concentration range of dsDNA contaminations (Lambda DNA) were present. The conditions of this test were as follows: apparatus, Infinite F200 multifunction microplate reader; dye, SYBR Gold; Ex 492 nm, Em 550 nm; slit width of filters, both were 10 nm.

### Method selectivity for mismatches

Method selectivity was assessed by using one to three base mutations of H1N1 complementary ssDNA (Table 1). All assay conditions were the same as described in “parallel analysis” section. The instrument parameters of this test were as follows: Ex 492 nm, Em 550 nm (dye, SYBR Gold); slit width of filters, both were 10 nm.

### Evaluation of the strategy using 4% tissue homogenate samples

In order to illustrate the practical use of this method, 4% pork muscle tissue homogenate (final concentration) was used to simulate the reality, and 50 pM H1N1, CaM, and HCV virus ssDNAs in the tissue homogenate have been directly detected. The procedures are as follows:

10% pork muscle tissue homogenate (prepared by HB) were denatured for 10 min at 95°C. Then, it was centrifuged for 5 min at 5000 rpm, and the supernatant was transferred to a clean test tube. 20 µL 0.5 µM target ssDNA HB solution and 40 µL 0.5 µM complementary ssDNA HB solution were pipetted into a well and hybridized in 80 µL 10% pork muscle tissue homogenate. For control groups, 40 µL 0.5 µM complementary ssDNA HB solutions were replaced by HB; for blank control, we used 60 µL HB instead of all DNA solutions in the experiment. After incubating for 5 min at room temperature, 40 µL S1 nuclease solution (36 U, prepared according to the instruction: 0.1 µL S1 nuclease per 20 µL 1× reaction buffer, purchased from Takara, Dalian, China) was added and incubated at room temperature for 30 min. Finally, 20 µL 10× SYBR Gold was added and incubated for another 5 min, and then the fluorescent intensities were recorded (final concentration of tissue homogenate is 4%). The excitation (Ex) and emission (Em) wavelengths settings of Infinite F200 multifunction microplate reader were 492 nm and 550 nm, respectively, and the slit width of filters both were 10 nm. Experiments were repeated three times.

### Statistical Analysis

All statistical analyses were performed using IBM SPSS Statistics (Version 19) software. Data are expressed as the mean ± standard deviation. Group comparisons were carried out by using standard t-test, and the significance level was measured at less than 0.05.

## Results and Discussion

**Figure 1:**
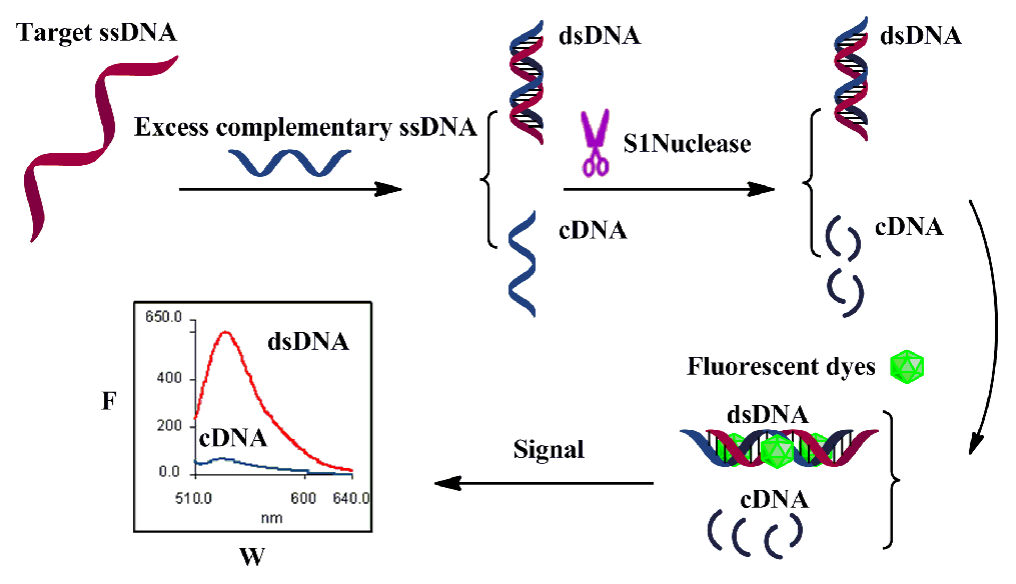
Illustration of the label-free ssDNA detection strategy for detecting sequence-specific ssDNA target with its complementary ssDNA, S1 nuclease and DNA fluorescent dyes.

The simple detection strategy is presented in Fig. 1. In this strategy, S1 nuclease plays an important role which degrades single-stranded nucleic acid or cleaves dsDNA at the single-stranded region caused by a nick, gap, mismatch or loop. This ability makes it feasible to cleavage the possible small ssDNA ‘bubbles’ surrounding the mismatch site induced by the complementary ssDNA [10], [11], which gives the strategy a discrimination capability of sequence mismatches. Additionally, S1 nuclease could also eliminate excessive complementary ssDNA or other single-stranded nucleic acid contaminations to decrease non-specific fluorescent background, therefore enhance the sensitivity and practicability of the proposed method.

**Figure 2:**
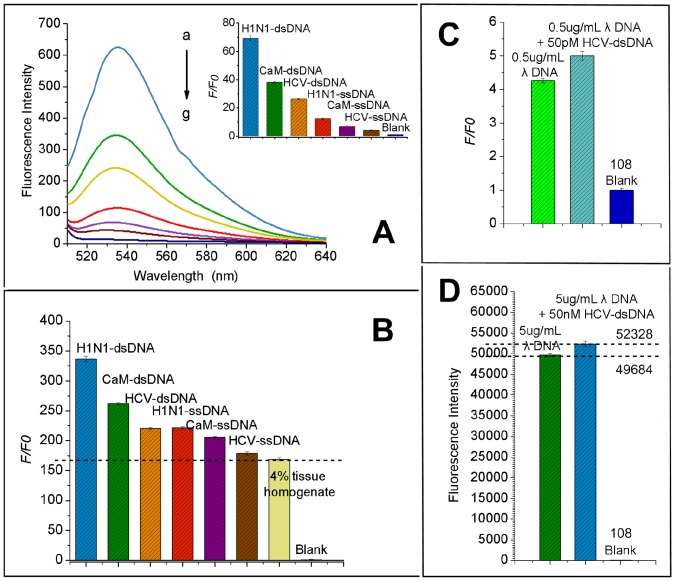
Detection results of Sequence-specificity ssDNA in different surroundings. (A). Fluorescence intensities of three viruses ssDNA and their hybridized products after S1 nuclease-mediated cleavage using SYBR Gold (1×). The concentration of target ssDNAs are 50 nM, and their complementary ssDNAs are 100 nM. a, H1N1-dsDNA; b, CaM-dsDNA; c, HCV-dsDNA; d, H1N1-ssDNA; e, CaM-ssDNA; f, HCV-ssDNA; g, blank. (B). Fluorescence intensities of three viruses ssDNA and their hybridized products in 4% pork muscle tissue homogenate after S1 nuclease-mediated cleavage using SYBR Gold (1×). The concentration of target ssDNAs are 50 nM, and their complementary ssDNAs are 100 nM. (C). Fluorescence intensities of 50 pM HCV-ssDNA and its hybridized products in 0.5 µg·mL^−1^ Lambda DNA standard solution after S1 nuclease-mediated cleavage using SYBR Gold (1×). (D). Fluorescence intensities of 50 nM HCV-ssDNA and its hybridized products in 5 µg·mL^−1^ Lambda DNA standard solution after S1 nuclease-mediated cleavage using SYBR Gold (1×).

As can be seen from Fig. 2A, the hybridization of virus ssDNA with the complementary ssDNA shows significantly increasing of fluorescent intensity and gives a high F/F0 value (F0 represents the fluorescent intensity of solution blank containing S1 nuclease, HB solution and DNA dyes, and F represents the fluorescent intensity of the solution as described above upon addition of target ssDNA, or target ssDNA and its complementary sequence). Since SYBR Gold exhibits >1000-fold fluorescence enhancement after binding to double- or single- stranded DNA or RNA [13], it still has excellent discrimination ability whatever there was 50 nM excessive complementary ssDNA with the S1 nuclease addition. This discrimination ability also enabled it to detect the samples in complex conditions directly. Fig. 2B is the corresponding results of the practical use. In 4% pork tissue homogenate, samples (50 nM) were determined in order to show in parallel the anti-interference capacity of the method, and the test reproduced the result of Fig. 2A. Besides that, a concentration range of lambda dsDNA was used as external contamination to investigate its discrimination ability. HCV as the lowest fluorescence efficiency sample in this test (Fig. 2A, Fig. 2B), was employed as the target to find this limit. Fig. 2C and Fig. 2D are the results of this test. 50 pM HCV-ssDNA (containing 0.5 µg·mL^−1^ Lambda DNA as external dsDNA contamination) induced the fluorescence increase were shown in Fig. 2C, and result was significant (P<0.01); also, 50 nM HCV-ssDNA can be detected in 5 µg·mL^−1^ Lambda DNA standard solution, P<0.01. Since most of the currently used methods for ssDNA detection (PCR, probe hybridization, etc.) need an extraction and purification step, this strategy would be a good alternative choice to simplify the detection of pathogens in the field and in the clinic.

**Figure 3:**
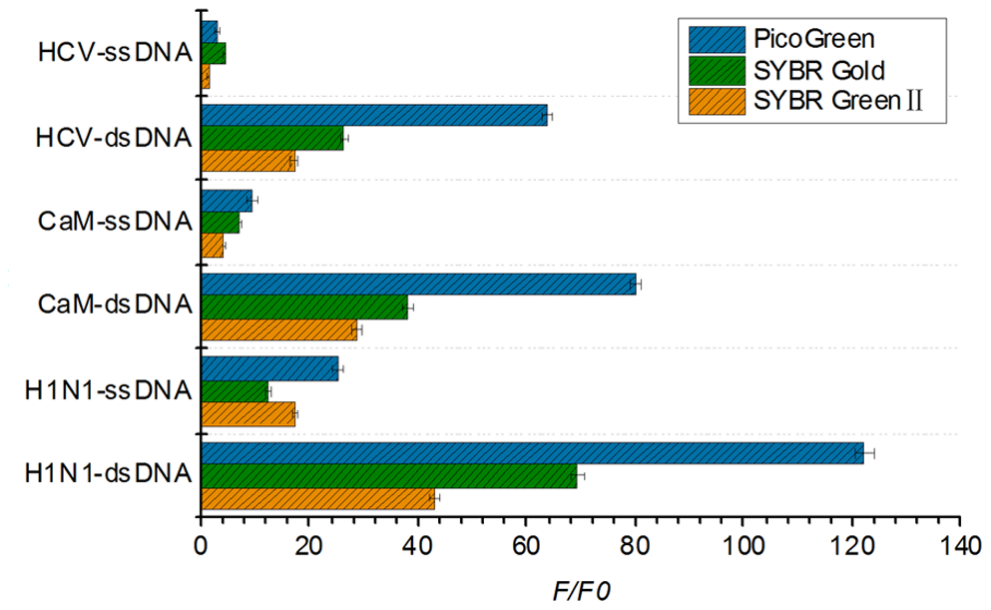
F/F0 values of three viruses and three types of dyes obtained from the proposed strategy. The concentrations of target ssDNA and their complementary ssDNAs are 50 nM and 100 nM respectively, and the concentration of dyes is 1×. F0 represents the fluorescent intensity of solution blank containing S1 nuclease, HB solution and DNA dyes, and F represents the fluorescent intensity of the solution as described above upon addition of target ssDNA, or target ssDNA and its complementary sequence.

Moreover, two other different fluorescent DNA-dyes SYBR Green II and PicoGreen were tested the universality of this approach. PicoGreen represents dsDNA specific dye and SYBR Green II represents ssDNA dye [14] while the above mentioned SYBR Gold is a universal nucleic acid fluorescence dye for double- or single-stranded DNA or RNA. As shown in Fig. 3, it is interesting to find that all of three DNA dyes can detect target viruses ssDNA efficiently at the presence of S1 nuclease while PicoGreen gives the highest quantum yield upon binding to dsDNA or ssDNA [15].

Compared with Zeng's ssDNA detection method using complementary ssDNA without S1 nuclease [12], the proposed method in this study shows high sensitivity, specificity and universality. It could not only detect H1N1 virus sequence but also CaM and HCV. The F/F0 value of HCV, which was reported that could not be detected by using ordinary nucleic acid fluorescent gel stains in Zeng's articles [12], [16], could be detected in our research by all of the three dyes, and their F/F0 values are 17.05 (SYBR Green II), 26.43 (SYBR Gold), and 63.60 (PicoGreen). From the data, we could find that even for HCV detection, all of the F/F0 values of three dyes are higher than H1N1 in previous report [12]. We considered it as a result of S1 nuclease-mediated cleavage of redundant complementary ssDNA and nonspecific hybridization.

**Figure 4:**
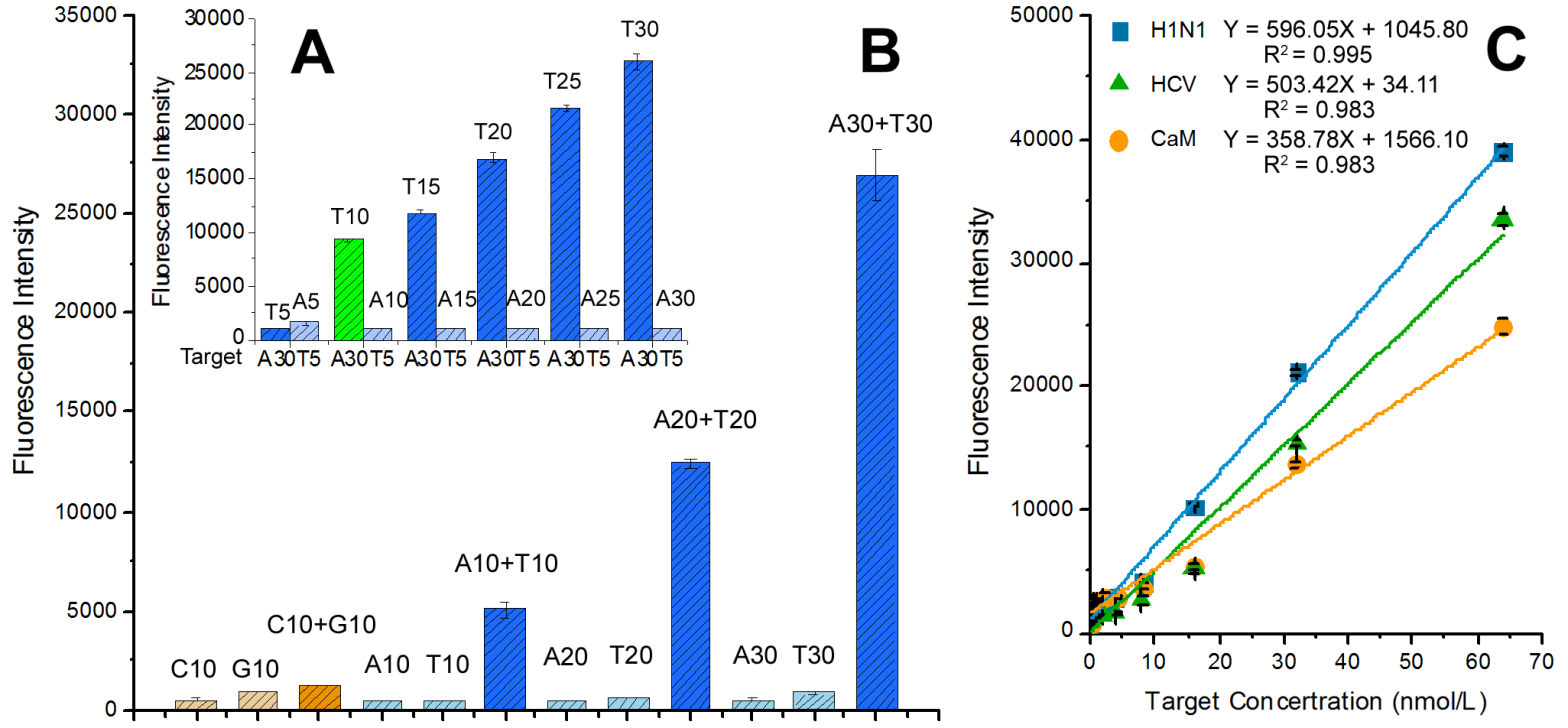
Fluorescence intensity of oligonucleotides hybridized products with 1× PicoGreen after S1 nuclease-mediated cleavage. The concentration of oligonucleotides and their complementary oligonucleotides is 50 nM and 100 nM respectively. (A) The effects of lengths of target ssDNA on fluorescence intensity. (B) The effects of nucleotide composition of target ssDNA on fluorescence intensity. (C) Linearity of the proposed method (three virus ssDNA). Plots of fluorescence intensity versus different concentrations of target ssDNA hybridized products (linear range, 50 pM–64 nM).

The effects of nucleotide composition and lengths of target ssDNA on the fluorescence intensity were investigated using different lengths oligonucleotides (5, 10, 15, 20, 25, and 30 bases) with different bases (A/T and G/C). From Fig. 4B, we could find that, compared to A/T, the G/C oligonucleotides only produced a low fluorescence signal that means PicoGreen has the nucleotide selectivity and A/T base pair playing a dominant role in the fluorescence intensity increasing. In addition (Fig. 4B), the fluorescence intensity increased significantly with increasing target ssDNA lengths. As a result of S1 nuclease-mediated cleavage, the fluorescence intensities of single-strand oligonucleotides were almost been eliminated while the fluorescence signals of their hybrid products were very strong.

Poly (T) and poly (A) with different lengths was used to further study the effect of ssDNA length on the fluorescence intensity. As shown in Fig. 4B, When different lengths of oligonucleotides were hybridized (A30 hybrid with T5, T10, T15, T20, T25 and T30 respectively or T5 hybrid with A5, A10, A15, A20, A25 and A30 respectively), the increasing fluorescence intensity has positive correlation with the lengths of hybridized dsDNA as a result of S1 nuclease-mediated cleavage of redundant ssDNA, like “A30+T5” and “A5+T5”. That means the complementary sequence should have at least the same length as the target ssDNA to obtain high sensitivity by using the proposed method. The results further confirmed the S1 nuclease role in sensitivity and specificity of the proposed method as assumed above.

From Fig. 4A and 4B, it was shown that even A5/T5 hybrid products produced a fluorescence intensity of about 1000; the A10/T10 gave nearly 5000 fluorescence intensity. In Fig. 4A, the fluorescence intensity was increased by 8.27 folds from five A/T base pairs to ten A/T base pairs (A5/T5 to A30/T10, Fig. 4A, green bar). Back to the HCV ssDNA we detected above, though there are only seven A/T, HCV ssDNA still gave high fluorescence intensity and could be easily detected. The results suggested that the A/T number in target ssDNAs is the key of the sensitivity of the strategy and more than five A/T is needed to use the proposed method.

To further demonstrate the proposed method sensitivity and specificity, a series of concentrations of three virus ssDNA were mixed with 5 ng·mL^−1^ Lambda DNA which was used as external dsDNA contamination. The fluorescent intensities were recorded by subtracting the background fluorescence of Lambda DNA. The linear range and sensitivity for three virus ssDNA were obtained as shown in Fig. 4C. The S1 nuclease-mediated cleavage can increase the fluorescent signal-to-noise ratio by reducing nonspecific background fluorescence and the measured fluorescence intensities are linearly proportional to the concentrations of the target ssDNA. As showed in Fig. 4C, for H1N1, a linear relationship of Y = 596.05X+1045.80 was found in the range of 50 pM–64 nM with R^2^ = 0.995 and a detection limit (defined as mean of blank control +3 times the standard deviation) of 17.91 pM (n = 3); for CaM, Y = 358.78X+1566.10 with a liner range from 50 pM to 64 nM, R^2^ = 0.983 and a detection limit of 45.23 pM; and for HCV, Y = 503.42X+34.11 with a liner range from 50 pM to 64 nM, R^2^ = 0.983 and a detection limit of 40.51 pM. The results were also further evidences that the proposed method could specifically detect the target ssDNA even in contaminant-ridden samples. The present detection limits were much lower than that of the reported one (H1N1, 480 pM, and the other two viruses could not be quantitatively detected by common DNA dyes) [12].Thus, the high sensitivity and specificity of the strategy may fulfill the requirement for the sensitive and quantitative evaluation of most of ssDNAs.

**Figure 5:**
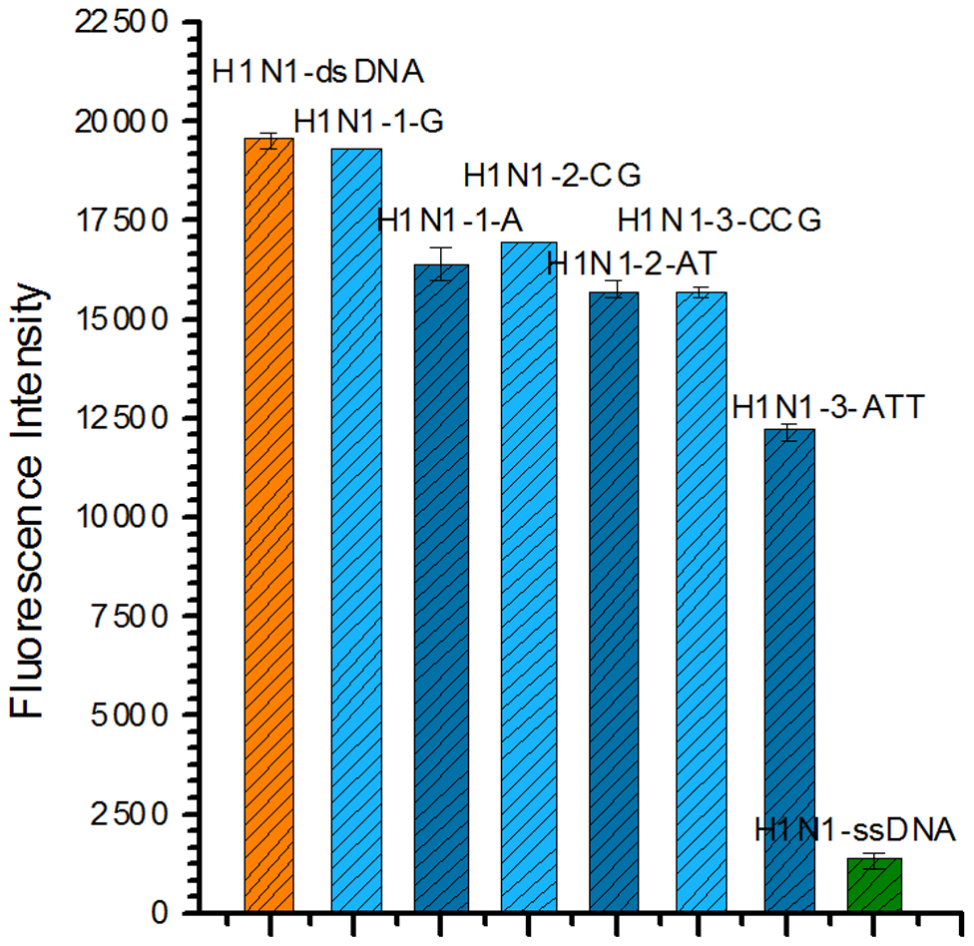
Method selectivity for mismatches (H1N1, 1×SYBR Gold). H1N1-1-G, H1N1-1-A: one base mismatch and the mismatched base is “G”, “A”, respectively. H1N1-2-CG, H1N1-2-AT: two bases mismatches and the mismatched bases are “CG”, “AT”, respectively. H1N1-3-CCG, H1N1-3-ATT: three bases mismatches and the mismatched bases are “CCG”, “ATT”, respectively. The concentration of all target ssDNAs is 50 nM, and their complementary ssDNAs is 100 nM.

Additionally, the method selectivity for mismatches was assessed too. By using one to three base mutations of H1N1 complementary ssDNA (Table 1), it exhibited a good selectivity for mismatches. From Fig. 5, we could find a downtrend from perfect matched H1N1-dsDNA to three base pairs mismatches, though, compare to H1N1-dsDNA a “G” variation in the sequence could not give out a significant difference (P = 0.132). However, the difference of H1N1-dsDNA and an “A” variation (or two base pairs mismatches) were significant (P<0.01). In reality, SNP-s or other variations could potentially affect the detection of the target DNA and might lead to underestimation. Alternatively, incorrect pairing to non-specific contaminating DNA (that might occur in PCR too) might also lead to overestimation of the target DNA. This experiment presented the signal changes when there are one to three mismatches between target and probe DNA. It provided a good proof for the possible use of this method in related fields in the future.

## Conclusions

In this paper, we have presented a simple, sensitive, and specific label-free strategy for ssDNA detection by using its complementary ssDNA, S1 nuclease and nucleic acid fluorescent dyes. The introduction of S1 nuclease enhanced the fluorescent signal-to-noise ratio by clearing away other single-stranded nucleic acid contaminations, redundant complementary ssDNA, and possibly mismatched double strand DNAs. By taking advantage of the super fluorescence efficiency and well sequence specificity of this method, it can detect target ssDNA in complex samples directly and exhibits a high sensitivity and specificity towards target ssDNA (e.g. H1N1, with a detection limit of 17.91 pM and two base pairs mismatches). Moreover, the strategy is facile and cost-effective, and can provide a universal platform for sensitive detection of various ssDNA and RNA in the field and in the clinic.
